# A case of I-cell disease (mucolipidosis II) presenting with short femurs on prenatal ultrasound and profound diaphyseal cloaking

**DOI:** 10.1259/bjrcr.20150420

**Published:** 2016-07-28

**Authors:** Thomas Capobres, Gauravi Sabharwal, Brent Griffith

**Affiliations:** Department of Radiology, Henry Ford Health System, Detroit, MI, USA

## Abstract

A 28-year-old G3 P1 SAB1 female with no prior health concerns was found to have a foetus with short femurs on prenatal ultrasound following an abnormal maternal serum screen result. Fluid obtained by amniocentesis revealed an elevated α-fetoprotein level with absence of an acetylcholinesterase band and normal male karyotype (46,XY). Follow-up ultrasound 3 weeks later again demonstrated short femur lengths, but no other abnormalities. At birth, the child was noted to have multiple dysmorphic features, including short humeri and femurs, coarse facial features, retrognathia and yellowish hypertrophic gums in addition to hyperbilirubinaemia and thrombocytopenia. Radiological studies demonstrated bony demineralization with profound diaphyseal cloaking in the long bones. Genetic testing diagnosed I-cell disease.

## Background

Mucolipidosis II (I-cell disease) is a lysosomal storage disorder caused by deficiency of *N*-acetylglucosamine-1-phosphotransferase. Nearly all lysosomal hydrolases are elevated in the plasma and body fluids of affected individuals because of the failure of targeting lysosomal acid hydrolases to the lysosomes. It is characterized by the presence of cytoplasmic inclusions in cultured fibroblasts (I-cell phenotype).^1^,^2^ There is a significant amount of variability in the disorder in terms of age of onset, tissue/organ involvement and radiological findings.^2^ Despite the heterogeneous manifestations, the disorder typically presents between 6 and 12 months of age with clinical symptoms that include marked growth deficiency, coarse facial features, severe skeletal abnormalities, developmental delay and gingival hyperplasia.^3^–^5^ The presenting radiological findings at that time are often of severe dysostosis multiplex, although these findings vary depending on the stage at which they are found.^1^,^2^

Recognizing the potential prenatal and early postnatal findings of I-cell disease allows earlier diagnosis. Given the universally fatal prognosis, early diagnosis is crucial in helping affected families plan and cope with the outcome. We present a case with both prenatal and early postnatal findings of the disease.

## Case report

### Clinical presentation

**Figure 1.**
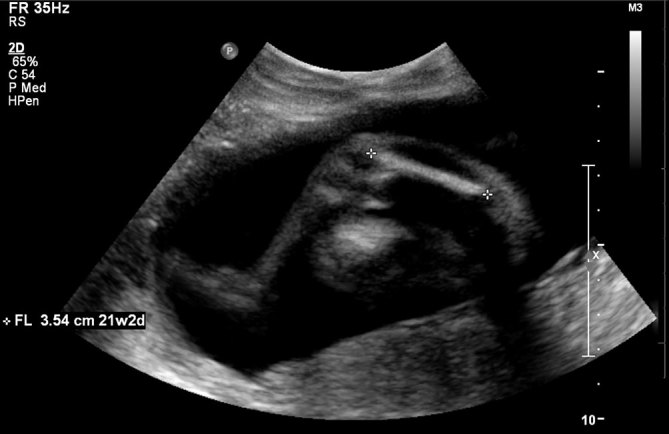
Prenatal ultrasound at 22 6/7 weeks gestational age demonstrates short femurs measuring 3.5 cm (< 2.5%).

A 28-year-old G3 P1 SAB1 female with no prior health concerns was found to have an abnormal integrated maternal serum screen indicating a 1 : 7 risk for trisomy 18. Foetal ultrasound at 19 weeks gestation revealed short femurs (< 2.5%) and an otherwise normal examination. A multidisciplinary approach was taken, including a genetics consult. The family history was notable for one nephew with “weak bones”. The patient and her husband reported that they were of Yemeni ancestry and distantly related. A recommended amniocentesis was performed, which showed increased α-fetoprotein at 2.26 MoM and absence of an acetylcholinesterase band. Chromosome analysis revealed a normal male karyotype (46,XY). Follow-up ultrasound at 22 weeks gestation again demonstrated short femurs, measuring 3.5 cm (< 2.5%) (Figure 1). Biparietal diameter, head and abdominal circumference measured between the 39th and 55th percentiles. The patient chose to forgo further follow-up with genetics, and the remainder of the pregnancy was otherwise uneventful.

The baby was born by C-section at 37 4/7 weeks gestation owing to foetal decelerations. Upon delivery, the infant was found to have hoarse cry, weak reflexes and low tone. Multiple dysmorphic features were discovered, including short humeri and femurs; bowed lower legs; narrow chest; large ear lobes; retrognathia; yellowish hypertrophic gums and a low, flat palate; hypertrichosis of the bilateral temporal region; and light hair colour that was atypical for his ethnic background. He exhibited diffuse patchy ecchymoses on the trunk and persistent thrombocytopenia as well as hyperbilirubinaemia. Echocardiogram showed a small atrial septal defect and a large patent ductus arteriosis. The infant also experienced respiratory distress, requiring continuous positive airway pressure ventilation.

### Investigations/imaging findings

**Figure 2.**
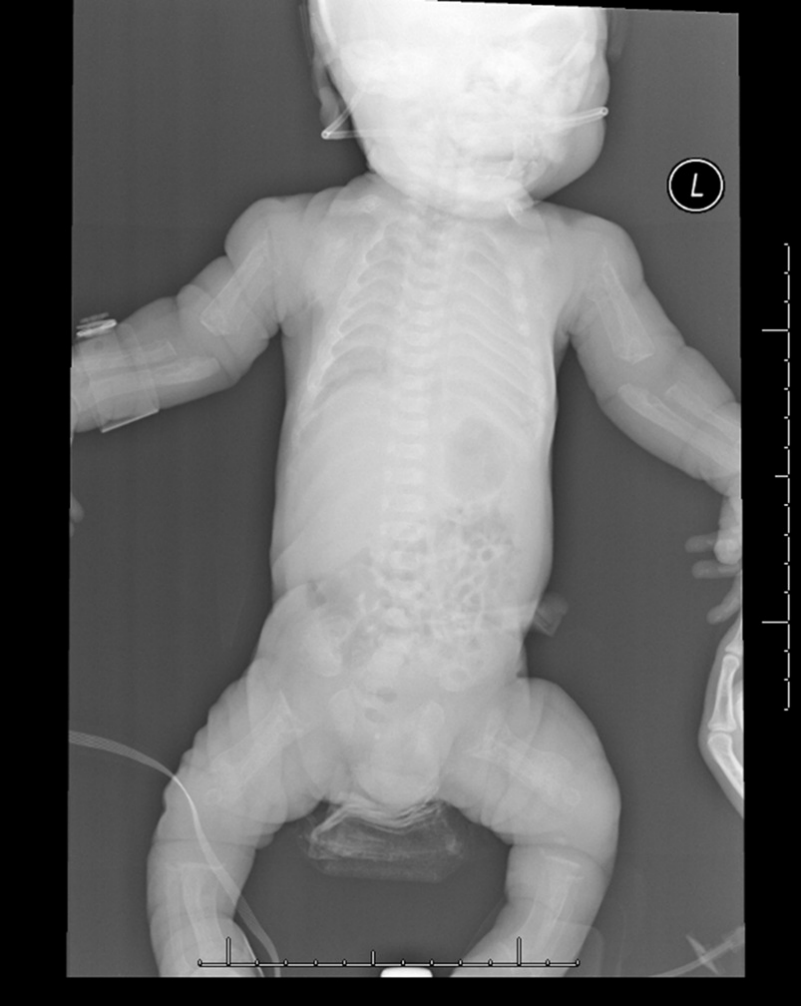
Skeletal survey demonstrates diffuse demineralization of bony structures and relatively short humeri and femora.

**Figure 3.**
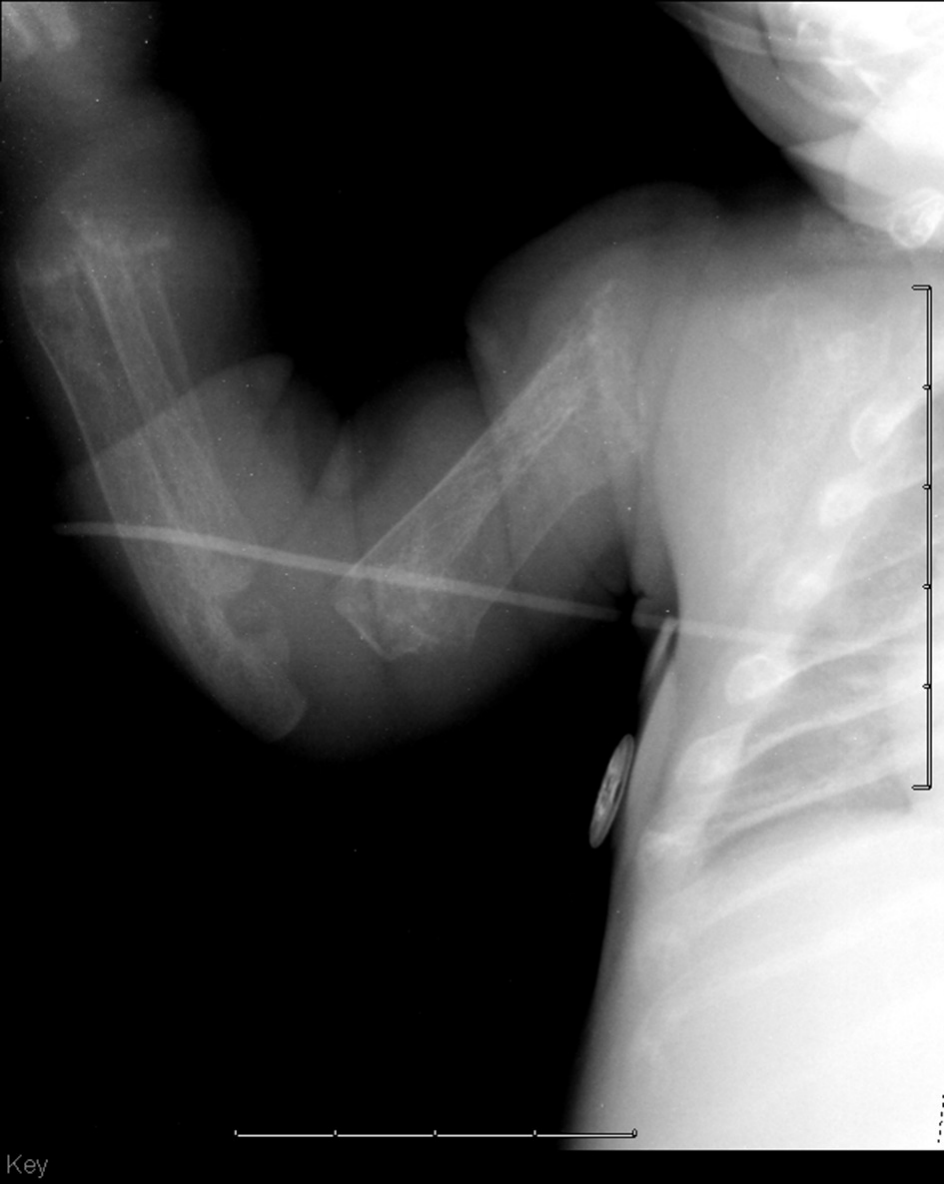
The humerus demonstrates poorly formed and irregular appearing proximal metaphyses and profound diaphyseal cloaking. The forearm demonstrates unusual bowing of the distal ulna and radius with metaphyseal cupping.

**Figure 4.**
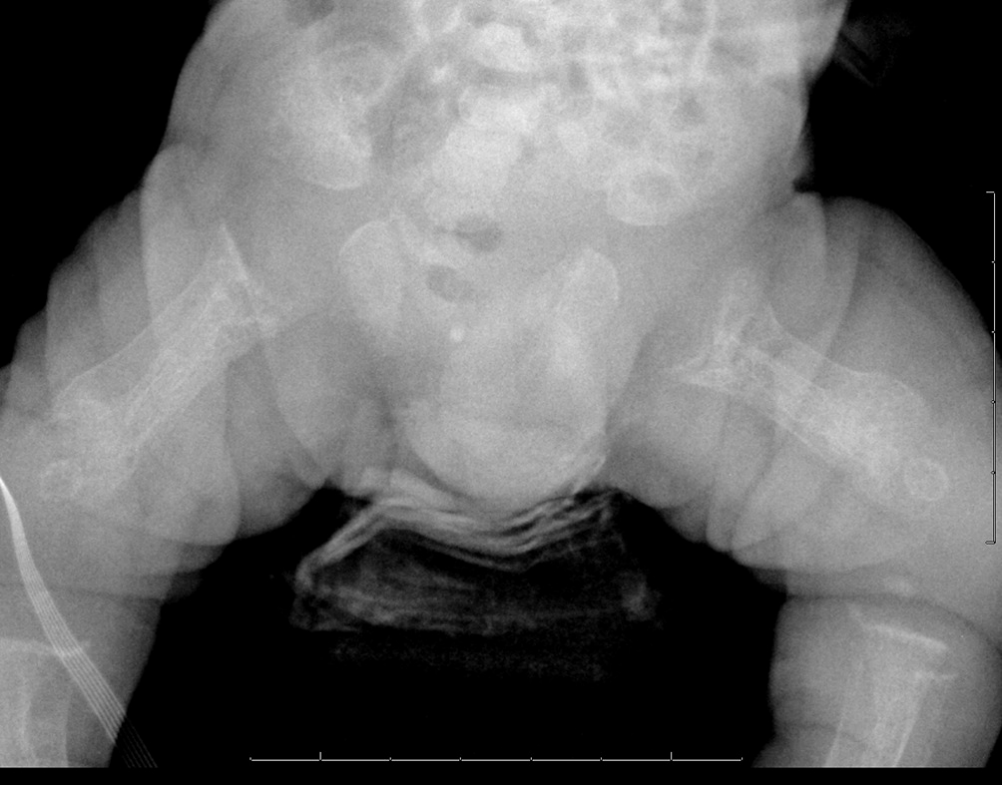
The pelvis demonstrates poorly formed iliac bones with flattening of the acetabular roofs and irregular appearing proximal femoral metaphyses with periosteal cloaking.

Radiological investigation at that time revealed the following:

- diffuse demineralization of bony structures (Figure 2)
- profound diaphyseal cloaking of the long bones (Figures 2 and 3)
- relatively short humeri and femora (Figure 2)
- poorly formed and irregular appearing proximal humeral and femoral metaphyses (Figures 2 and 3)
- thickened and poorly formed clavicles (Figure 2)
- thickened and shortened ribs with an abnormally increased cardiothoracic ratio (Figure 2)
- poorly formed iliac bones with flattening of the acetabular roofs (Figure 4)
- unusual bowing of the distal ulna and radius with metaphyseal cupping (Figure 3)
- thickening of the proximal phalanges and minimal narrowing at the proximal aspect of the metacarpal bones.

Genetics service was consulted. Tests revealed elevated levels of multiple plasma and leukocyte lysosomal hydrolases, consistent with a diagnosis of I-cell disease. *GNPTAB* gene analysis revealed homozygous c.376_379delTTAG deletion mutations. This deleterious mutation has not been reported previously in individuals with I-cell disease.

### Outcome

The patient was eventually discharged to the care of hospice and passed away at 5 weeks of age. Additional information regarding the case was limited as an autopsy was declined by the family.

However, the parents did follow-up with genetics several months later for future family planning. When the wife became pregnant again later that year, chorionic villous sampling was obtained at 10 weeks gestational age and sent to test for I-cell disease. Uridine diphosphate-*N*-acetylglucoseamine-1-phosphotransferase enzyme activity was low, consistent with I-cell disease. Soon after obtaining these results, the family chose to terminate the pregnancy at 15 weeks. Chorionic villous testing was again obtained during a fifth pregnancy, and testing revealed that this child was neither affected nor a carrier of the abnormal *GNPTAB* gene. This child was born at full term without any health concerns.

## Discussion

I-cell disease is a rare lysosomal storage disorder that typically remains undiagnosed until later in the first year of life, as facial features coarsen, growth failure becomes increasingly apparent and the Hurler-like clinical picture develops.^3^,^5^ It is caused by mutations in the *GNPTAB* gene, located on chromosome 12, and inherited in an autosomal recessive manner. In the appropriate clinical setting, such as when a family history of the disorder is known, foetal I-cell disease can be suggested sonographically by the demonstration of abnormally shortened long bones and intrauterine growth retardation.^6^ Other sonographic features that have been described in the literature include periosteal cloaking of the femora and humeri and transient maxillary defects.^7^ In our case, the only abnormality found on prenatal ultrasound was bilateral short femora (< 2.5%). No other signs of intrauterine growth retardation or additional sonographic abnormalities were present. In the absence of a known family history, prenatal diagnosis of I-cell disease remains difficult as many other conditions can present with similar findings.

The postnatal features of I-cell disease are more classic and have been better characterized in the literature. A study of nine cases of the disease by Lemaitre et al^1^ in 1978 discussed the specific radiological features of the disorder and distinguished I-cell disease from other storage disorders.

In their study, the authors divided the radiological manifestation of I-cell disease into two groups—Stage A, an *early stage;* and Stage B, a *late stage.*^1^ Stage A includes the neonatal period and the first 2 months after birth, while Stage B starts after the age of 4 months.^1^

The *early stage* is typically characterized by a storage osteopathy with bone structural and modelling abnormalities, stippling of the epiphyses and elements of a storage disorder.^1^ These signs evolve during the *intermediate stage* with disappearance of the bone structural abnormalities and the signs of a storage disease becoming more pronounced.^1^ In the late stage, the skeletal manifestations are of an early and severe dyostosis multiplex.^1^ The *late stage* findings, while apparent at 4–6 months, continue to become more obvious as the patient grows older.

Despite the *early stage* findings described, I-cell disease typically goes undiagnosed until later in the first year of life. In the study by Lemaitre et al,^1^ seven of the nine cases were diagnosed after the neonatal period. More recently, David-Vizcarra et al^8^ attempted to better define the natural history and early osteodystrophic features of I-cell disease. They described neonatal radiographic findings in 15 cases of I-cell disease that were similar to those seen in neonatal hyperparathyroidism.^8^ Later studies have also presented cases manifesting similar radiographic findings with markedly elevated alkaline phosphatase and parathyroid hormone levels as well as normal serum calcium levels suggesting severe secondary hyperparathyroidism.^9^ However, the aetiology of severe secondary hyperparathyroidism in I-cell disease is unclear.^8^,^9^ In their study, David-Vizcarra et al^8^ concluded by emphasizing the need for further studies regarding the pathophysiology of osteodystrophy in I-cell disease and the need for more definitive characterization of the early clinical manifestations.^8^

Our case demonstrates many of the features described in the *early stage* of the disease, including diffuse bony demineralization, shortened long bones with periosteal apposition, poorly formed metaphyses with cupping, thickened and shortened ribs and clavicles, flattened acetabular roofs and rectangular metacarpals with coning of the proximal ends. However, it is the dramatic degree of periosteal apposition, or diaphyseal cloaking, seen in the long bones that sets this case apart from previously reported cases.

Diaphyseal cloaking is thought to be due to increased remodelling of the bone. It is typically unilamellar, although it has been described in some cases as general periostitis. In earlier studies, it has been described as a poorly defined contour or a double outline image. In addition, it is typically seen in the diaphyseal region of the bones.

In the current case, the degree of cloaking was more dramatic than in previously reported cases of I-cell disease. In addition, the cloaking did not have the appearance of simple periosteal apposition, but demonstrated a more solid appearance. Adding to the uniqueness of the case, the cloaking was not confined to the diaphyses but rather extended to the metaphyseal regions.

Another unique aspect of our case is the persistent thrombocytopenia, which has not been previously described in reported cases of I-cell disease presenting in the neonatal period. Could this also relate to the marked degree of periosteal cloaking in our patient? Subperiosteal haemorrhage, as seen in cases of infantile scurvy, can appear similar to diaphyseal cloaking, although in the former case this appearance is not confined only to the diaphyseal regions.^10^ Perhaps the dramatic degree of periosteal cloaking in our case represents excessive subperiosteal new bone formation in the setting of subperiosteal haemorrhage either in addition to, or even as opposed to, sequelae of secondary hyperparathyroidism. Unfortunately, an autopsy was not performed on our patient. Future studies with note of any associated thrombocytopenia as well as pathologic correlation are needed to investigate this hypothesis.

Given the universally fatal prognosis of I-cell disease, early diagnosis is crucial in helping affected families cope with the outcome. While prenatal sonographic features of the disorder are non-specific, recognizing them in the appropriate clinical setting is paramount in making the diagnosis. When the prenatal diagnosis is not made, radiologists can play an important role in suggesting the possible diagnosis. We have presented a case with prenatal short femurs and dramatic neonatal osteodystrophic features. In addition to supporting the classically defined radiological manifestations of early I-cell disease, we also offer a unique hypothesis that early stage finding of diaphyseal cloaking could also relate to subperiosteal haemorrhage. Further studies are needed to better understand the pathophysiology behind the characteristic early osteodystrophy seen in I-cell disease. Larger case series are also necessary to fully characterize the early manifestations of the disease. With continued work in these areas, earlier diagnosis of this universally fatal disease may be possible.

## Learning points

1. I-cell disease is a rare lysosomal storage disorder that typically goes undiagnosed until later in the first year of life, as facial features coarsen, growth failure becomes increasingly apparent and a Hurler-like clinical picture develops.
2. Findings on prenatal ultrasound are variable but may include abnormally shortened long bones, intrauterine growth retardation, periosteal cloaking of the femora and humeri, and transient maxillary defects.
3. The *early stage* from the neonatal period through 2 months after birth is typically characterized by storage osteopathy with bone structural and modelling abnormalities, stippling of the epiphyses and elements of a storage disorder. The *late stage* starts after the age of 4 months and is typically characterized by skeletal manifestations of an early and severe dyostosis multiplex.
4. The pathophysiology of the early osteodystrophy seen in I-cell disease is currently poorly understood. Better defining the early clinical and radiological manifestations is key to achieving earlier diagnosis of this universally fatal disease.

## Consent

Despite exhaustive efforts undertaken by the researchers to contact the family of the deceased patient, they could not be reached, and thus informed consent could not be obtained. However, the case report has been sufficiently anonymized so as to prevent harm to the patient's family as confirmed by Dr Ezhuthachan, Head of Neonatology at Henry Ford Hospital.
